# Porous Nanostructured Gadolinium Aluminate for High-Sensitivity Humidity Sensors

**DOI:** 10.3390/ma14227102

**Published:** 2021-11-22

**Authors:** Corneliu Doroftei, Liviu Leontie

**Affiliations:** 1 Research Center in Environmental Sciences for the North-Eastern Romanian Region (CERNESIM), Science Research Department, Institute of Interdisciplinary Research, Alexandru Ioan Cuza University of Iasi, Bulevardul Carol I, Nr. 11, 700506 Iasi, Romania; lleontie@uaic.ro; 2 Faculty of Physics, Alexandru Ioan Cuza University of Iasi, Bulevardul Carol I, Nr. 11, 700506 Iasi, Romania

**Keywords:** gadolinium aluminate, sol-gel self-combustion, structural properties, capacitive/resistive humidity sensors

## Abstract

This paper presents the synthesis of gadolinium aluminate (GdAlO_3_), an oxide compound with a perovskite structure, for applications as a capacitive and/or resistive humidity sensor. Gadolinium aluminate was synthesized by the sol-gel self-combustion method. This method allowed us to obtain a highly porous structure in which open pores prevail, a structure favorable to humidity sensors. Most of the materials studied as capacitive/resistive humidity sensors have significant sensitivities only with respect to one of these types of sensors. In the case of the studied gadolinium aluminate with *p*-type electric conductivity, the relative humidity of the air has a significant influence on both capacitive and resistive types of electric humidity sensors. The capacity increases about 10,000 times, and the resistance decreases about 8000 times as the relative humidity increases from 0 to 98%. The investigated gadolinium aluminate can be used successfully to obtain high-sensitivity capacitive and/or resistive humidity sensors.

## 1. Introduction

Humidity is a physical parameter that describes the amount of water vapor found in the atmosphere. It is an extremely important factor in many industrial sectors [1,2]. Thus, for the measurement and control of humidity, it is necessary to develop high-performance humidity sensors using environmentally friendly materials and processes.

Due to low costs and good thermal and chemical stability, ceramic sensors are in the spotlight of current research [3]. The operation principle of ceramic humidity sensors is based on two methods of measuring humidity: the *resistive electrical method*, based on the variation in the effective electrical conductivity of a material in the presence of water vapor, and the *capacitive electrical method*, based on the variation in the effective dielectric constant of a porous dielectric material in the presence of water vapor.

Among the oxide compounds, perovskites (simple or doped, prepared through various methods) occupy a very important place in terms of applications concerning metal–air batteries [4], supercapacitors [5,6], and humidity/gas sensors [7,8,9,10,11].

Zou et al. [12] studied a series of simple perovskites—SmFeO_3_, NdFeO_3_, LaFeO_3_—in the form of nanofibers synthesized by the electrospinning method as resistive humidity sensors within the range of a relative humidity (RH) of 11–95%. All the studied humidity sensors exhibit optimal working frequency of 100 Hz at which the sensors display good linearity and high response. Compared with other sensors, the SmFeO_3_ nanofiber humidity sensor shows the best sensitivity, and the impedance variation is more than five orders of magnitude, with the relative humidity changing from 11% to 95%.

Josephine et al. [13] studied lanthanum chromite doped with magnesium ions (LaMg*_x_*Cr_1−*x*_O_3−*δ*_ where *x* = 0.0, 0.2, 0.4, 0.6, 0.8 and 1), obtained by the sol-gel method, for resistive humidity sensor applications. Measurements of resistance versus relative humidity within the range of 5–98% were performed, and the humidity-sensing factors (S*_f_* = R_5%_/R_98%_) were calculated. Chromite doped with magnesium ions (*x* = 0.4) had the highest humidity-sensing factor of 21,407, while chromite without magnesium (*x* = 0) had the lowest sensitivity factor, namely 27.27. The response and recovery characteristics for magnesium-doped (*x* = 0.4) chromite exhibited a good linearity and a very narrow hysteresis loop.

Upadhyay and Kavitha [14] have performed a study concerning the applications of barium stannate doped with La ions (Ba_1−*x*_La*_x_*SnO_3_, where *x* = 0.0–0.1), synthesized by the classical method (ceramic), for both capacitive and resistive humidity sensor applications. This study shows that the working frequency has very little influence on the resistivity of the sensor, while the capacity is very much influenced. As the value of the relative humidity of the environment increases from 10 to 95%, the resistivity of barium stannate doped with La ions changes considerably compared to its capacity.

A very important class of perovskites from an applicative point of view is that of aluminates doped with lanthanide elements [15,16,17,18,19].

This work presents the synthesis of gadolinium aluminate (GdAlO_3_) perovskite, as well as the investigation of this compound for capacitive and/or resistive humidity-sensing device applications. Gadolinium aluminate was synthesized via the sol-gel self-combustion method with polyvinyl alcohol (PVA) as a colloidal medium [20,21,22]. The method used is superior to other classical methods and allows obtaining perovskite structures with high porosity in which open pores predominate, forming channels that facilitate the absorption and desorption of vapors.

## 2. Materials and Methods

Nanocrystalline GdAlO_3_ perovskite was synthesized by the sol-gel self-combustion method [22] using gadolinium nitrate, aluminum nitrate, ammonium hydroxide and PVA as a colloidal medium. The solutions of gadolinium and aluminum nitrates in stoichiometric amounts were mixed with a solution of PVA, resulting in a colloidal solution. An amount of ammonium hydroxide solution is added until a pH of 8 is obtained, thus resulting in a soil of metal hydroxides. By drying this soil followed by self-combustion, a nanocrystalline powder is obtained, which was then calcined at 500 °C for 30 min, in order to eliminate possible residues of an organic nature. One part of the calcined powder was heat treated for 1 h at 900 °C, while the other part was heat treated for 7 h at 1000 °C.

To obtain information on the values of the transition temperatures during the formation of perovskite-type phases, the calcined powder obtained through self-combustion has been characterized appealing to thermogravimetric and differential thermal analysis (TG-DTA) techniques, using Netzsch STA 449 F1 Jupiter apparatus (NETZSCH-Gerätebau GmbH, Selb, Germany). Information on the structural properties of the heat-treated powders was obtained by X-ray diffraction (XRD) measurements using a PANALYTICAL X’ PERT PRO MPD diffractometer (Malvern Panalytical, Morvin, UK) with CuK_α_ radiation (*λ* = 1.54251 Å). The surface morphology of the heat-treated powders and the elemental surface composition were examined by scanning electron microscopy (SEM-EDX), using a JEOL-200CX microscope equipped with an EDX module. The values regarding the pore volume, the specific surface area and the average particle size were determined by the Brunauer, Emmet and Teller (BET) method, with a Nova-2200 instrument (Quantachrome, Boynton Beach, FL, USA).

The sensing element (both capacitive and resistive) was made of as-synthesized pressed powders in the form of discs with a diameter of 17 mm and a thickness of 2 mm, to which porous silver metal electrodes were deposited on both surfaces using the “screen printing” method.

The values of the capacity and electrical resistance of the sensor element were determined using an impedance analyzer (Agilent 4294A, Santa Clara, CA, USA) that operates in the frequency range of 40 Hz–110 MHz.

For the measurement of humidity, the sensing element was inserted into a thermostat chamber maintained at 25 °C under different humidity conditions. The value of 0% RH was obtained by using dry calcium chloride (CaCl_2_), while the values of RH between 11% and 98% were obtained by using saturated solutions of some salts [23]. The response time of the sensor element to humidity variation was obtained by registering the capacitance or resistance changes when the RH varied between two values in both directions (absorption/desorption).

## 3. Results and Discussions

### 3.1. Structure and Morphology

**Figure 1:**
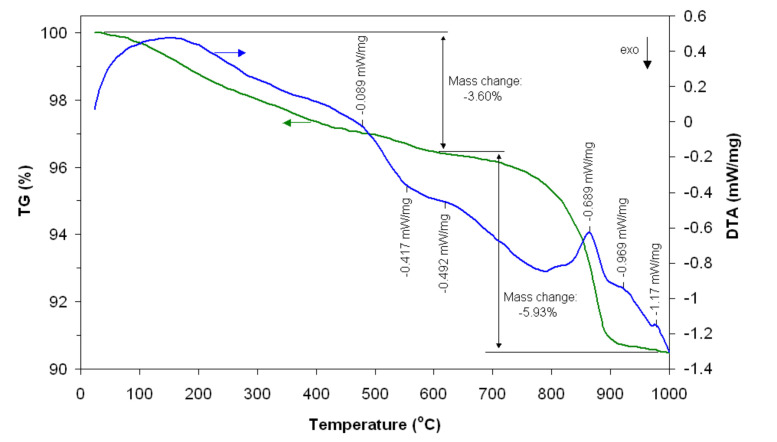
TG-DTA curves of gadolinium aluminate (before heat treatment).

Samples obtained through self-combustion have been characterized using thermal analysis. In Figure 1, the TG-DTA curves are shown, where one can notice that the sample mass decreases with increasing temperature up to 480 °C due to the loss of water, volatile substances and some crystallization reactions. At a temperature of around 560 °C, the DTA curve indicates the presence of an exothermal peak that may be due to the combustion reaction of some organic compounds, as well as to the residual carbon, with a total weight loss of about 3.60%, followed by an endothermic peak located at 620 °C, which can be attributed to certain crystalline phases of the material. At temperatures over 620 °C, one can observe some endothermic peaks, while the bigger ones are situated around the values of 870 °C and 925 °C, where one can notice a pronounced mass loss (5.93%). Around the temperature value of 980 °C, one can observe another endothermic peak, with a constant mass tendency, which suggests the formation of the crystalline structure of gadolinium aluminate.

By this method, gadolinium aluminate can be obtained at a much lower temperature (1000 °C) than required using a classical method [17,24,25]. Cizauskaite et al. [16] have obtained the GdAlO_3_ perovskite without secondary phases by means of heat treatment at 1000 °C over 10 h, and also through the sol-gel preparation method by using metal nitrates as the starting material, while the colloidal medium was obtained through several intermediate reactions, and the powder was obtained through slow calcination.

**Figure 2:**
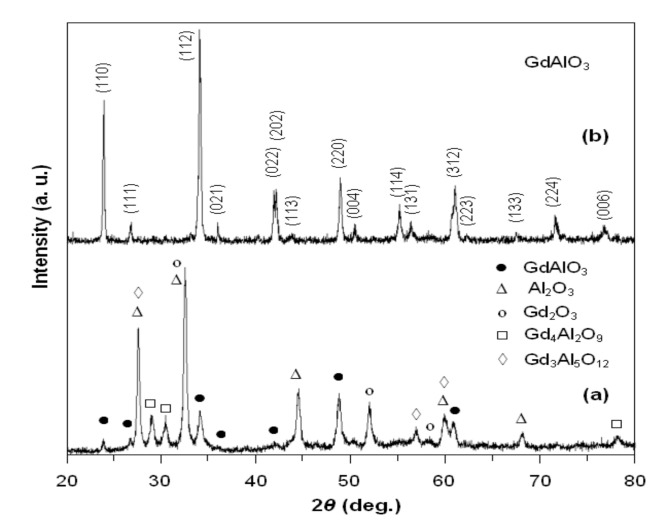
The X-ray diffractograms for gadolinium aluminate heat treated at 900 °C/1 h (**a**) and 1000 °C/7 h (**b**).

The X-ray diffraction pattern of the powder treated at 900 °C for 1 h (Figure 2a) shows the appearance of crystalline phases of GdAlO_3_ and of some secondary phases (Al_2_O_3_, Gd_2_O_3_, Gd_4_Al_2_O_9_, and Gd_3_Al_5_O_12_). In addition, the X-ray diffractogram for the powder heat treated at 1000 °C for 7 h (Figure 2b) reveals the presence of perovskite phases, without other secondary phases.

According to the PDF card No. 46–395, the gadolinium aluminate obtained exhibits an orthorhombic symmetry (Pbnm space group).

**Figure 3:**
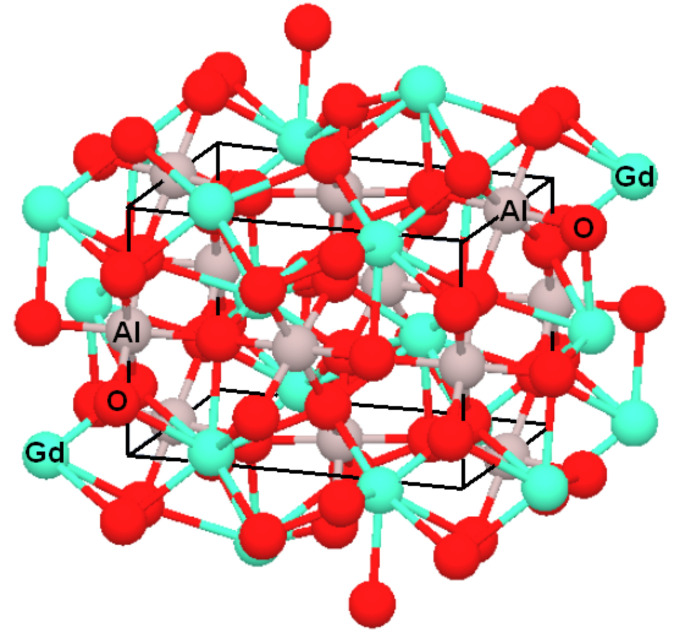
Spatial structure and unit cell of GdAlO_3_ perovskite.

The values obtained for the lattice parameters (*a* = 5.250 Å; *b* = 5.302 Å; *c* = 7.447 Å) of the powder heat treated at 1000 °C/7 h are in accordance with the values found by other authors [17]. These values are also in agreement with the theoretical values obtained by Persson [26]: *a* = 5.285 Å; *b* = 5.371 Å and *c* = 7.511 Å, which also represented the spatial structure of the unit cell, where Gd^3+^ ion is bonded in an eight-coordinate geometry to eight O^2−^ ions, and Al^3+^ ion is bonded to six O^2−^ ions to form a corner-sharing AlO_6_ octahedra (Figure 3).

**Figure 4:**
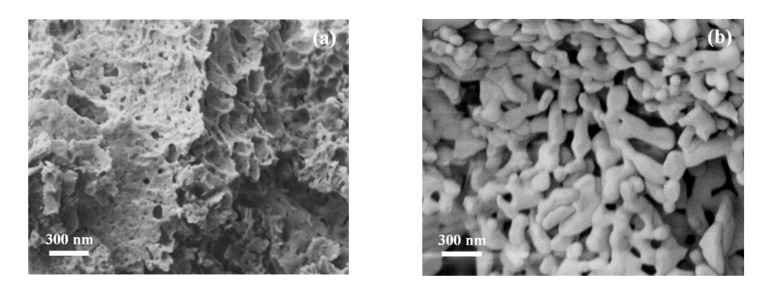
SEM micrographs for gadolinium aluminate heat treated at 900 °C for 1 h (**a**) and at 1000 °C for 7 h (**b**).

From the SEM micrographs, one can notice a major change in the sample microstructure with the increase in heat treatment parameters from 900 °C/1 h (Figure 4a) to 1000 °C/7 h (Figure 4b). After heat treatment of the powder at 900 °C for one hour, a prevalent amorphous porous structure is revealed, in which nanoparticles are also visible. Making a comparison with the data obtained from XRD patterns, one can confirm that the material contains some GdAlO_3_ crystal phases, along with numerous secondary phases. After heat treatment of the powder at 1000 °C for 7 h, a major change in morphology is revealed. The entire mass of the material is crystallized. This confirms the data obtained from XRD and TG-DTA analyses, which indicated the formation of a perovskite structure without secondary phases.

The grain sizes determined through the linear intercept method have an average value between 70 and 100 nm. A structure with accentuated porosity can be observed, in which open pores predominate, forming channels that facilitate the adsorption and desorption of vapors (Figure 4b).

**Figure 5:**
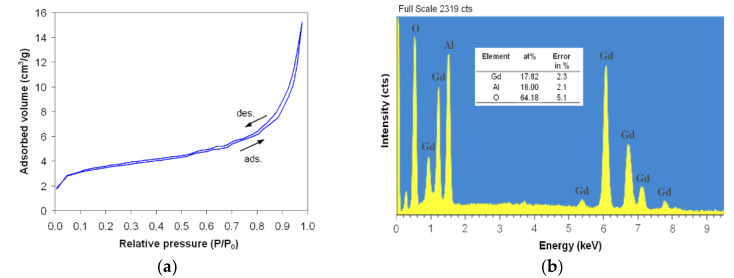
N_2_ adsorption/desorption isotherms (**a**) and EDX spectrum with the analyzed elements (**b**) for gadolinium aluminate powder, heat treated at 1000 °C for 7 h.

From the BET analysis for gadolinium aluminate with a crystal structure without secondary phases (heat-treated powder at 1000 °C/7 h) valuable information was obtained concerning the specific surface area (*S*_BET_), the average particle size (*D*_BET_) and the total pore volume (*V*_BET_). Figure 5a shows the N_2_ adsorption/desorption isotherms at 77 K. According to the IUPAC classification [27], isotherms are type IV with a small H_3_-type hysteresis loop. The inflexion point of isotherms reflects the stage of complete monolayer coverage, when multilayer adsorption begins to take place. From BET analysis, the specific surface area, *S*_BET_ = 10.0 m^2^/g, the average particle size, *D*_BET_ = 81.0 nm, and the total pore volume, *V*_BET_ = 0.0018 cm^3^/g, were obtained. The value of the average particle size obtained from the BET analysis falls within the value ranges obtained from the SEM analysis.

Energy-dispersive X-ray spectroscopy (EDX) provides an accurate determination of the atomic concentrations of different elements present in the surface region of the samples. Figure 5b shows the EDX spectrum and the elemental analysis for the heat-treated powder at 1000 °C for 7 h. It can be seen that the composition of the sample is the same as that of the nominal one, ABO_3_, i.e., the A/(A + B) or B/(A + B) ratio has a value of about 0.5, proving a homogeneous distribution of the elements in the sample (A is Gd at. % and B is Al at. %).

### 3.2. Sensor Properties

For the sensor element obtained from the heat-treated powder at 1000 °C for 7 h, the capacitance and resistance were measured depending on the relative humidity of the environment as well as working frequency.

**Figure 6:**
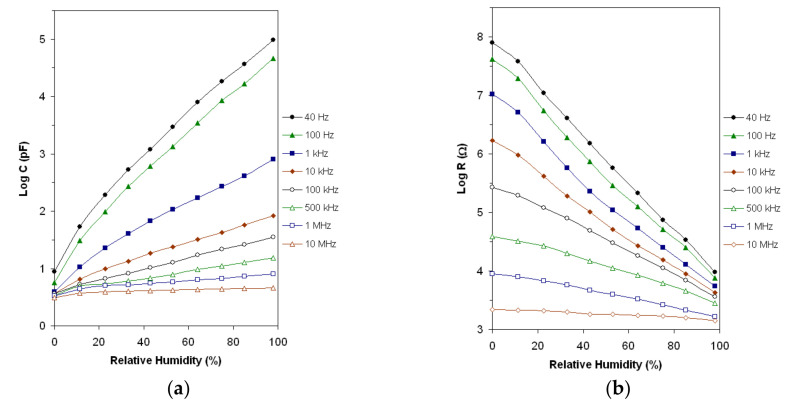
The capacitance (**a**) and resistance (**b**) vs. RH characteristics at eight working frequencies in 40 Hz–10 MHz frequency range.

In Figure 6a,b, the log C and log R, respectively, vs. RH characteristics are shown at room temperature (25 °C) for the frequency range of 40 Hz–10 MHz. The characteristics exhibit good linearity in logarithmic scale for all working frequencies and their slope decreases with the increase in the working frequency.

Humidity sensitivity (*S*) can be calculated using the equation [28]:

(1) $$S=\frac{M_{max}}{M_{min}}-1$$

with *M_max_* and *M_min_* representing the capacitances (C) or resistances (*R*) measured for the maximum and minimum values of the relative humidity interval, respectively.

**Figure 7:**
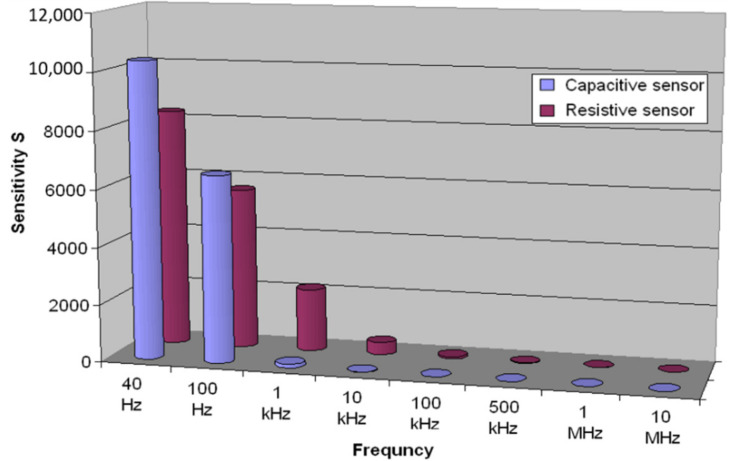
The sensitivity of both sensor regimes, capacitive and resistive, depending on the working frequency in the range of 0–98% RH.

In Figure 7, the sensitivity for both sensor regimes, capacitive and resistive, for eight working frequencies in the range 0–98% RH is shown. Regarding the capacitive sensor, the highest value of sensitivity, around 10,000, is reached for the working frequency of 40 Hz and decreases significantly to the value of 125 for the working frequency of 1 kHz. Regarding the resistive sensor, the highest value of sensitivity, around 8000, a slightly lower value than in the case of the capacitive sensor, is obtained at a frequency of 40 Hz and decreases exponentially to the value of 0.6 for the frequency of 10 MHz.

Regarding the capacitive humidity sensor, when the sensor material is exposed to humidity, the water molecules are absorbed and the material presents a leak conduction (γ) [29]. The electrical capacity (*C*) of the material can be expressed as follows [30,31]:

(2) $$C=\varepsilon^{*}\times C_{0}=\left(\varepsilon_{r}-i\frac{\gamma }{\omega \varepsilon_{0}}\right)\times C_{0}$$

where ε^*^, *C*_0_ and *ε_r_* represent the complex dielectric constant, capacitance and relative dielectric constant of an ideal capacitor, respectively; *ω* represents the angular frequency, *γ* represents the conductance and *ε*_0_ represents the permittivity of free space. This equation shows that the value of the capacity decreases with the increase in the working frequency as well as with the increase in the humidity value. Moreover, conductance increases with the increase in humidity, which implies the increase in the capacitance with the increase in humidity as a function of the angular frequency [1,30,31].

In the case of low working frequencies, the capacity variation with humidity can be explained by the phenomena of adsorption and absorption of water molecules, and their effect on the variation in the system capacity formed by porous material and water [32,33].

When several layers of water molecules are adsorbed on the material surface, they will be linked together by a single hydrogen bond, leading to increased mobility of the molecules and, therefore, to an increase in the complex dielectric constant. If the absorption of liquid water also takes place, the water molecules are extremely mobile and the relative dielectric constant becomes the maximum, corresponding to the water [34].

At high working frequencies, energy losses do occur due to the ionic conductivity that is achieved by hopping transfer of protons between adjacent hydroxyl groups [35]. The energy losses lead to the decrease in the complex dielectric constant and therefore to the decrease in the slope of log *C* vs. RH curve.

Regarding the resistive humidity sensor, when the sensor is exposed to humidity, the electrical conduction nature can be of two types: ionic, in which the sensor resistivity decreases with the increasing RH due to absorption and water vapor condensation in capillary pores; and electronic, in which the sensor resistivity depends on the semiconductor type (*n* or *p*).

The sensor resistivity shows a decrease when the water molecules are absorbed due to absorption and water vapor condensation in the open pores. In this case, the sensor manifests an ionic conduction, controlled by H^+^ ions resulting from the water from the sensor material surface. We performed Hall measurements and determined that the gadolinium aluminate studied has *p*-type semiconductor behavior. Therefore, we can also talk about a *p*-type conduction, where the water molecules adsorbed on the semiconductor surface play the role of electron acceptor. This manifestation of electronic conduction supplements the ionic conduction in the studied material.

The maximum difference between the RH values at absorption/desorption for the same value of capacity or resistance was defined as hysteresis. In the practice of sensor applications, this value must be as low as possible.

**Figure 8:**
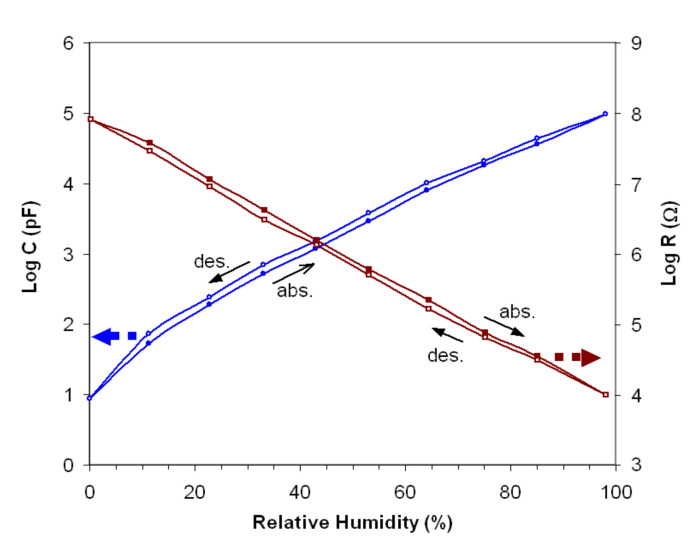
The absorption and desorption curves of both capacitive and resistive sensor.

The gadolinium aluminate studied for humidity capacitive/resistive sensor application exhibits good reversibility (small hysteresis) in the low-frequency range, at increasing and decreasing RHs, within the investigated humidity range. In Figure 8, the absorption and desorption curves, log *C* and log *R* vs. RH, are shown, in the range of 0–98% RH and at the optimal working frequency (40 Hz). The hysteresis value is very low (0.3–3%) for both types of sensors on the entire RH range. Displaying a small hysteresis, when the relative humidity of the environment will decrease, desorption of water molecules will be achieved with lower additional energy.

Another important factor for a humidity sensor consists of the response time. When the sensor at a certain humidity value is exposed to a higher humidity value, we can define a response time to absorption. When returning to the initial value, we can define a response time to desorption. The time required for the response value to reach 90% of the variation value for absorption and desorption, respectively, was considered the response time.

The gadolinium aluminate was studied as the humidity capacitive/resistive sensor was investigated regarding the absorption and desorption response time, for the RH range of 0–98% and the frequency range of 40 Hz–10 MHz. The sensor element in both regimes, both as a capacitive sensor and as a resistive sensor, had a response time of around 45 s for absorption and around 60 s for desorption at a humidity variation of about 11%. It was found that the response time is not considerably influenced by the working frequency for both regimes of the sensor element (capacitive/resistive).

**Figure 9:**
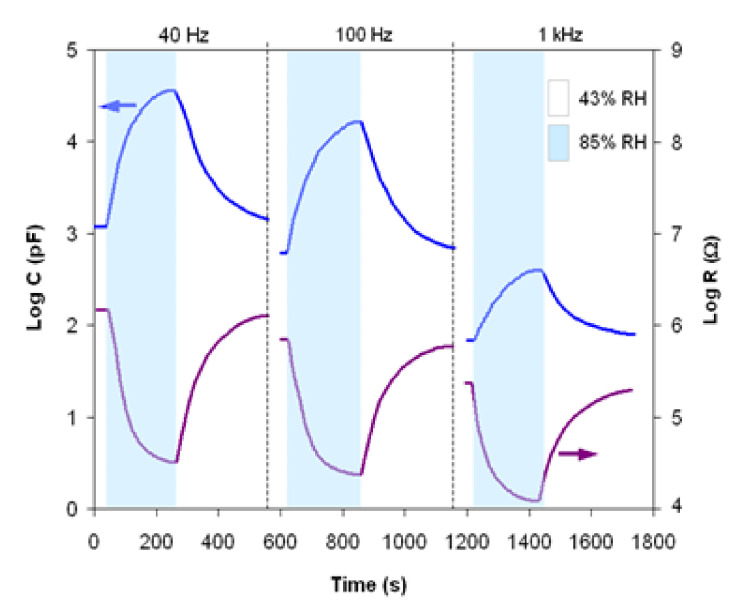
The response time characteristics for three working frequencies.

The response time characteristics at room temperature (25 °C) for the sensor element (capacitive/resistive) within an RH range between 43% and 85% (a usual working domain for humidity sensors) at frequencies of 40 Hz, 100 Hz and 1 kHz are shown in Figure 9. Both capacitive and resistive sensor regimes exhibit a response time of about 180 s for absorption (43–85% RH) and about 230 s for desorption (85–43% RH) and are not considerably influenced by the working frequency. The response time to desorption was found to be slightly longer than to absorption, due to different water vapor adsorption/desorption rates, determined by the microporous structure of the sample, in particular by pore size and distribution.

## 4. Conclusions

Gadolinium aluminate with a perovskite structure, synthesized by the sol-gel self-combustion method, has been investigated for applications as a capacitive and/or resistive humidity sensor.

The sensor’s electric capacity increases about 10,000 times within a 0–98% interval of relative humidity, and the electric resistance decreases about 8000 times within the same RH interval at the optimum working frequency of 40 Hz. Used as a capacitive sensor, its sensitivity diminishes quickly with the working frequency up to 1 kHz. Used as a resistive sensor, its sensitivity exponentially decreases up to the working frequency of 10 MHz.

The Log *C* and Log *R* vs. RH characteristics of the sensor show good linearity at all working frequencies. The sensor shows a small hysteresis (<3%) and a response time of around 180 s.

The investigated gadolinium aluminate can be used successfully to obtain high sensitivity capacitive or resistive humidity sensors.

## Data Availability

The data presented in this study are available on request from the corresponding author.
